# A Review of the Statistical and Quantitative Methods Used to Study Alcohol-Attributable Crime

**DOI:** 10.1371/journal.pone.0139344

**Published:** 2015-09-29

**Authors:** Jessica L. Fitterer, Trisalyn A. Nelson

**Affiliations:** Spatial Pattern Analysis and Research Lab, Department of Geography, University of Victoria, Victoria, British Columbia, Canada; Aichi Cancer Center Research Institute, JAPAN

## Abstract

Modelling the relationship between alcohol consumption and crime generates new knowledge for crime prevention strategies. Advances in data, particularly data with spatial and temporal attributes, have led to a growing suite of applied methods for modelling. In support of alcohol and crime researchers we synthesized and critiqued existing methods of spatially and quantitatively modelling the effects of alcohol exposure on crime to aid method selection, and identify new opportunities for analysis strategies. We searched the alcohol-crime literature from 1950 to January 2014. Analyses that statistically evaluated or mapped the association between alcohol and crime were included. For modelling purposes, crime data were most often derived from generalized police reports, aggregated to large spatial units such as census tracts or postal codes, and standardized by residential population data. Sixty-eight of the 90 selected studies included geospatial data of which 48 used cross-sectional datasets. Regression was the prominent modelling choice (n = 78) though dependent on data many variations existed. There are opportunities to improve information for alcohol-attributable crime prevention by using alternative population data to standardize crime rates, sourcing crime information from non-traditional platforms (social media), increasing the number of panel studies, and conducting analysis at the local level (neighbourhood, block, or point). Due to the spatio-temporal advances in crime data, we expect a continued uptake of flexible Bayesian hierarchical modelling, a greater inclusion of spatial-temporal point pattern analysis, and shift toward prospective (forecast) modelling over small areas (e.g., blocks).

## Introduction

Alcohol supply restrictions continue to relax across the globe, leading to increases in disease [1,2], dependency [3], injury [4,5], and crime [6–11]. Of particular concern, is the large proportion (~30%) of criminal offences committed while intoxicated [12–15]. For instance, researcher’s continue to demonstrate that, independent of socio-economic and demographic influences, higher alcohol access leads to greater rates of crime, including violent offences [16–18], disturbance [19], property damage [20], and drunk driving [21].

Modelling the relationship between alcohol consumption and crime can enable the coordination of preventative police patrolling and alcohol access restrictions. As such, health researchers are tasked with understanding how populations will respond to alcohol access and promotion [22]. Main questions include: how the change and distribution of alcohol price [23–25], hours of sales [26–28], establishment types [10,29–32], or consumption patterns [21,33] influence the rate of criminal offences. To accurately estimate alcohol consumption and alcohol policy effects on crime, data quality and selection of appropriate statistical methods are integral.

Advances in Global Positing Systems (GPS) and Geographical Information Systems (GIS) studies are increasing the use of detailed spatial units for alcohol-crime modelling (e.g., neighbourhoods, blocks, [32,34–36]) and results from longer times-series (ten years plus) are becoming available (e.g., [28]). With data increasing in spatial and temporal detail the likelihood of dependency between analysis units and time periods increases. If not explicitly addressed, autocorrelation (positive correlation of data between regions or time periods) can violate the assumptions of statistical modelling leading to clustered residuals and an artificial decrease in standard errors, such that dependence between data reduces the effective sample size (n) [37,38]. As a result, a growing suite of methods have emerged to model spatial and temporal structure across the crime-alcohol studies.

To date, other reviews have summarized the effects of alcohol exposure on crime [22,39–43], but not the methods used for estimation of the effects. The objective of our study was to evaluate data and the suitability of quantitative analysis strategies to model the effect of alcohol access/consumption on crime abundance by synthesizing current trends and highlighting methods keenly adapted to spatial effects modelling. The review is structured in a manner that first describes the selection of studies reviewed. Secondly, data characteristics, applied spatial units, and dataset structure are summarized. Finally, dominant statistical approaches are reviewed and critiqued, and new opportunities for data measurement and spatial analysis are discussed.

## Study Selection and Synthesis

**Fig 1:**
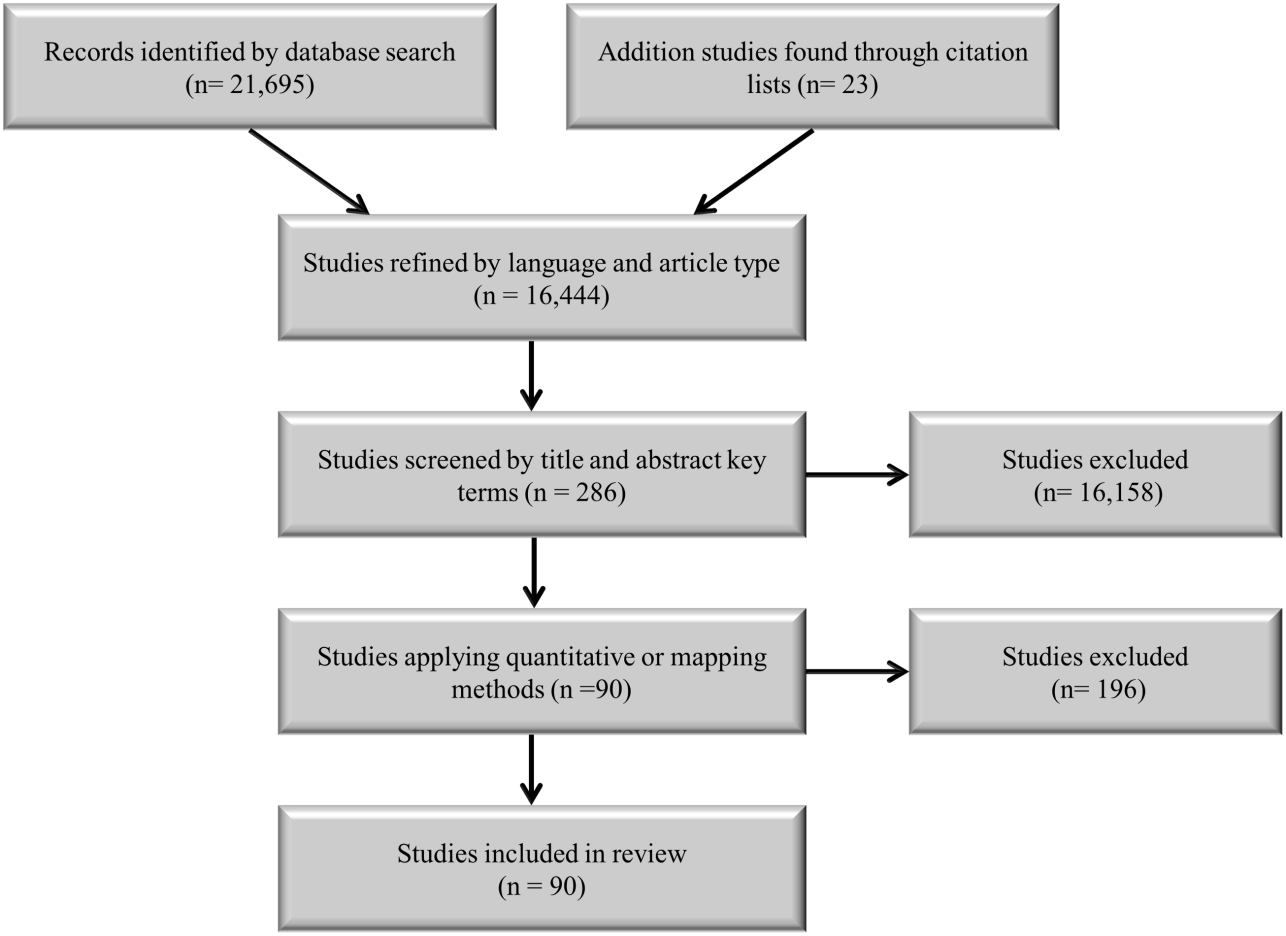
Publication selection steps.

We searched the alcohol-crime literature from 1950 to January 2014 using the Web of Science and Google Scholar databases. A list of key terms used singularly and combined with the following Boolean statement: (alcohol consumption OR binge drinking OR heavy drinking OR drinking patterns OR alcohol tax OR alcohol price OR alcohol cost OR alcohol outlet OR alcohol outlet density OR alcohol trading hours OR alcohol sales OR alcohol availability OR alcohol licensing OR on-premises OR off-premises OR bar OR pub OR hotel) AND (crime OR violent crime OR violence OR assaults OR domestic violence OR rape OR homicide OR interpersonal violence OR drinking and driving OR impaired driving OR drunk driving OR disturbance OR nuisance crime OR property crime OR amenity problems). Analyses that quantitatively evaluated or mapped the association between alcohol and crime were included (see Table 1 for search term descriptions and Fig 1 for publication selection steps).

**Table 1:** Search term descriptions.

Search Term	General description
Blood alcohol level	Percentage of alcohol contained in a person’s blood
Alcohol consumption	Ingestion of alcohol
Binge drinking	Drinking habits that lead to persons blood alcohol concentration to be 0.08grams or above [143]
Heavy drinking	Drinking five or more drinks at the same occasion five days out of thirty [143]
Drinking patterns	The frequency and amount of a person’s alcohol consumption
Alcohol tax	Government’s financial charge on alcoholic beverages
Alcohol price	The consumer’s price including cost and tax for purchasing alcohol
Alcohol cost	The consumer’s price before tax for purchasing alcohol
Alcohol outlet	On or off premises alcohol sales establishment
Alcohol outlet density	Measure of alcohol outlets per regional area standardize by population counts, roadways, or area
Alcohol trading hours	Permitted hours for alcohol sales
Alcohol sales	Days permitted to sell alcohol and gross profit received from alcohol sales
Alcohol availability	Population’s exposure to alcohol supply
Alcohol licensing	Permit allowing the sale of alcohol
On-premises	Establishment where alcohol consumption occurs within the building
Off-premises	Establishment where alcohol is purchased inside, but consumed outside
Bar	Establishment serving alcoholic drinks, sometimes dancing is encourage activity
Pub	Establishment serving alcoholic drinks and food
Hotel	Establishment offering housing that also serves alcoholic drinks and food
Crime	An action or omission that may be prosecuted by the government and is punishable by law
Violent	Using physical force to harm someone, a group, or something
Violence	Behaviour using physical force to harm someone, a group, or something
Assault	Physical attack against someone or something
Domestic violence	Violent or aggressive behavior between members of a home, usually between spouses or partners
Rape	Unlawful sexual acts or intercourse, with or without force, without the consent of the victim
Homicide	Deliberate killing of one person by another
Interpersonal violence	One person uses physical, mental, or financial power to control another person
Drinking and driving	Driving a motor vehicle after or during consuming alcohol
Impaired driving	Driving a motor vehicle while intoxicated
Drunk driving	Driving a motor vehicle while intoxicated by alcohol
Disturbance	Interruption of a settled environment
Nuisance crime	Minor crime that constitutes an injury, loss, or damage to a community rather than an individual
Property crime	Theft or destruction of someone’s personal belongings without force or threat of force
Amenity problems	Neighbourhood disturbance, litter, and noise

We calculated the number of countries represented across our sample and addressed if alcohol consumption was measured directly (e.g., blood alcohol level or survey admission of intoxication) or indirectly as measures related to exposure /use (e.g., alcohol price, hours of sales, or establishment access). Additionally, the frequency of different crime types and crime data sources were summarized. Spatial units were recorded and percent change in use before and after 2009 was calculated by subtracting the proportion of studies applying the analysis unit before 2009 from the proportion of studies applying the same unit after 2009. The results indicate an increasing or decreasing trend in unit application through time. Studies were then categorized by dataset structure including: cross-section (individual or regionally aggregated data collected at the same time), time-series (data collected over one region, but multiple time periods), panel (data collected over multiple spatial units and time periods), and intervention data (data indicating a change in alcohol exposure over time). After categorizing studies by structure, we summarized and critiqued methods used to estimate the effects of alcohol on crime including categories for: Autoregressive Integrated Moving Average (ARIMA) models, generalized linear regression (GLM), hierarchical and non-linear regression modelling (including spatial and temporal modelling), and finally a section for the regression trees, spatial, and temporal mapping.

## Results

### 3.1 Data

From the selections, 90 studies were included. Of selected studies, 56 were conducted in the United States and 16 in Australia, representing 80% of the sample (Table 2). The effects of alcohol consumption on crime were often measured as indicators related to alcohol use (89% of the studies), including: alcohol outlet counts or rates per region (n = 55), on-premises closing times (n = 10), alcohol tax (n = 7), volume of alcohol sold (n = 7), alcohol sales hours (n = 5), distance to alcohol outlets (n = 3), real price of alcohol (n = 3), or sale lock-outs (n = 2). The regional rates of alcohol outlet density exposure were calculated per 100,000 (n = 1), 10,000 (n = 3), 1,000 (n = 9), and 100 (n = 5) persons and as a density per square mile (n = 5) or roadway (n = 8). To a lesser extent, alcohol effects were measured directly from survey respondent consumption habits (n = 7) or blood alcohol levels (n = 3).

**Table 2:** Country study areas.

Country	Number of Studies
United States	56
Australia	16
Canada	5
Brazil	3
England	2
Sweden	2
Norway	2
New Zealand	1
Finland	1
Denmark	1
Scotland	1

Crime data included a variety of crime types and sources including aggregated crime categories (n = 8), violent offences (n = 70) drunk driving/crashes (n = 6), or nuisance crimes (n = 6), sourced from police records (n = 57), hospital admissions (n = 16), health statistics (n = 4), or surveys (n = 13). Five studies stratified police recorded data to peak alcohol drinking hours consisting of weekday and weekend evenings to reduce risk of spurious results [44–48].

### 3.2 Spatial units

Across the 90 studies selected, 68 studies used a spatial unit to measure crime or alcohol exposure. In most cases (n = 57, 83%), both crime and alcohol access data were aggregated to the same analysis unit. The remaining studies measured alcohol access across a larger spatial unit and related the regional alcohol exposure to crime reports associated with individuals. For example, state level alcohol taxes [49,50], and city [33], zip code [21,51], neighbourhood [20,52], census tract [6,29,53,54], campus [55] and police region [56] alcohol outlet density measures were used to estimate criminal incidences at the individual level.

Overall postal/zip codes (n = 11) and census tracts (n = 16) were the most commonly applied units. Analyzing trends before and after 2009 we found a decline in the use of larger state (-3%), postal code (-9%), city (-6%), municipality (-9%), and economic regions (-6%) and an increase in smaller police (9%), neighbourhood (9%), block (3%), block group (15%), and campus (3%) units (Table 3). The smaller unit studies were exclusive to North American.

**Table 3:** Applied analysis units counted by country, overall use before and after 2009, and the percent change in use after 2009. Percent change in use was calculated by subtracting the proportion of studies applying the analysis unit before 2009 from the proportion of studies applying the same unit after 2009.

Spatial Unit	Count Per Country								Overall Summary		
	United States	Australia	Canada	Brazil	United Kingdom	Norway	New Zealand	Finland	Count Before 2009	Count After 2009	% Change
Blocks	2		1						1	2	2.94
Block groups	7								1	6	14.71
Campuses	1								0	1	2.94
Neighbourhoods	5								1	4	8.82
Police Regions	2						1		0	3	8.82
Postal/Zip Codes	7	4							7	4	-8.82
Rural Areas		1							0	1	2.94
Census Tracts	16								9	7	-5.88
Municipalities	2			1					3	0	-8.82
Government Areas		2							1	1	0.00
Economic Regions					2				2	0	-5.88
Counties	2								1	1	0.00
Cities	2	1				1		1	3	2	-2.94
User defined	2								2	0	-5.88
States	5								3	2	-2.94

### 3.3 Dataset structure

Datasets were dominated by cross-sectional assessments (n = 54, 60%), eleven of which analyzed individual level crime data. Sixteen studies (18%) used time-series data to assess how alcohol exposure varied with crime across time, 15 of which monitored the change crime incidence after an alcohol policy intervention. Finally, 20 researchers (22%) conducted analyses on panel data to address how alcohol exposure varied with crime over time and space, in which five analyzed an alcohol policy intervention. Overall, the study of panel datasets was a trend of newer publications. Only four panel studies were published before 2009 [25,47,57,58].

### 3.4 Statistical approaches

#### 3.4.1 Autoregressive integrated moving average

Autoregressive Integrated Moving Average (ARIMA) models were selected for five of the 15 intervention time-series studies modelling the effects of on-premises alcohol establishments changes [59], alcohol tax reductions [60,61], change in outlet closing [28], and alcohol sales hours [62] on annual [59] and monthly incidence of assaults. Trends in the crime data were specified by three terms of the ARIMA model: the auto-regressive term (characterizes the temporal correlation in the series), the integrated trend (transforms the trend to stationarity), and the moving average (smoothes any random patterns or seasonal effects) [63]. Possible shifts in the incidence of crime were monitored using a dichotomous indicator variable (pre (0) and post (1) change).

#### 3.4.2 Generalized Linear Model

A large portion (n = 37, 41%) of the reviewed studies used generalized linear models (GLMs) to estimate the incidence of crime (outcome variable) as a function of the alcohol exposure and socio-demographic controls across intervention times-series (n = 10), cross-section (n = 23), panel (n = 3), and intervention panel (n = 1) datasets. GLMs are an extension of simple linear regression used to model crime as any member of the exponential family of distributions, conditional on the covariates, using a link function (e.g., log, logit, etc.). Expected crime distributions included binary (n = 7), multi-nomial (n = 3), count (n = 10), and rate (n = 17) outcomes.

Intervention time-series assessments used GLMs to analyze the change in monthly rates [44,64–67] and counts [18,27,68–70] of crime after on-premises outlet lock-out policy changes [18,65] or alcohol trading hour extensions [27,44,64,66–70]. In almost all cases, change in crime was assessed using a dichotomous intervention variable before and after the policy intervention period. Additional covariates were included in half of the studies to control for the impact of socio-demographics, other crimes, dry laws, polices force changes [65–67] and interactions among age, location and time of drinking [44] on crime. Seventy percent of the intervention studies used quasi control data to test that any change in criminal incidence was the effect of the alcohol policy intervention. Control data were integrated directly into the model by combining the alcohol policy intervention variable with a dichotomous area variable (study area verses control)(e.g., [70]).

More commonly, GLMs were applied to cross-section datasets estimating the effects of alcohol access on crime (n = 23). Eleven studies aggregated crime and alcohol measures to regional/spatial units to estimate the effects of increased excise tax [50] or alcohol outlet densities on crime rates [45,55,71–74] and counts [10,75–77], while controlling for area (spatial unit, fixed effect) and demographic characteristics. The remaining 12 cross-section studies estimated the probability of crime using individuals’ alcohol consumption data [13,78,79], alcohol access in the respondent’s regional area [20,52], or nested data investigating both consumption at the individual level and regional alcohol exposure [6,29,49,51,53,54,80].

Across panel analysis, fixed-effect GLMs (n = 4) were specified to estimate the impact of regional alcohol outlets [8,56,81] and change in alcohol tax [82] on monthly count [56], and annual [8,81,82] crime rates. The intervention in alcohol tax policy was monitored using categorical change variable, analogous with the intervention time-series regressions [82]. By specifying unit and time fixed effects GLM models maximize explaining the variance within units and years, limiting the possibility that differences between regions and time-periods will bias results (i.e., omitted variable bias).

#### 3.4.3 Hierarchical, non-linear models and extensions

An extension of GLMs are hierarchical models (generalized linear mixed models (GLMM), generalized additive models (GAM)), which were used to account for correlated errors, spatial patterns, and temporal trends (n = 40, 44%) across time-series (n = 1), cross-section (n = 27), panel (n = 8), and intervention panel (n = 4) datasets. Splines, data hierarchies, lagged variables, correlation terms, and random effects (slope and intercepts) addressed non-linear relationships, nested data structures, data dependency between units/time, and unexplained variance, respectively. Bayesian methods were also applied for inference in recent studies [30–32,35,83–86]. In contrast to frequentist techniques, Bayesian inference is conditional on both observed data (via the likelihood) and specified prior information for each model parameter to provide a joint posterior distribution for the model parameters. While the full posterior is generally not available in closed form, sampling methods (such as Markov Chain Monte Carlo) can be used to obtain samples from the marginal distributions, which are of primary interest.

The intervention time-series study [87] applied a negative binomial conditional autoregressive model (adjusted for age, sex, weekends, holidays, event days, government leadership) to estimate the influence of three different alcohol sales restrictions on the daily count of crime over four years. The autoregressive structure corrected for correlation between homicides in time (lagged counts of homicides from 7, 14, and 21 days). Intra annual trends (associated with alcohol policy changes) were modelled using marginal splines, and annual trends and seasonal changes in crime were captured using fractional polynomials and sine-cosine pairs (also known as Fourier terms), representing a thorough consideration of temporal oscillations in criminal activity.

Cross-sectional studies varied in model complexity to estimate the influence of alcohol expenditure [88], consumption frequency [21], or alcohol outlet density (n = 26) on the count (n = 4) and or rate (n = 23) of crime. Four studies published prior to 2004 used hierarchical regression to simultaneously estimate the influence of alcohol outlet density, socio-economic factors [89,90] and their interactions [91] on the rate of violent crime across municipalities, accounting for both individual level (frequency of drinking and driving with an intoxicated person) and city wide (alcohol outlet density) alcohol consumption influences on youth drinking and driving [33].

A larger portion (76%) of the cross-sectional studies conducted spatial regression to estimate crime rates within block groups (n = 5), neighbourhoods (n = 3), census tracts (n = 6), postal/zip codes (n = 5), government areas (n = 1), or user defined areas (n = 1) areas using spatial lag (SAR) [7,11,16,17,21,34,88,92–98], conditional autoregressive (CAR) [32,83], or spatial error (SEM) [84,92,94,99,100]. SAR, CAR and SEM models address spatial dependence within the outcome or exploratory variables (correlation of data between analysis units) to avoid spatially clustered residuals and biased coefficients [101]. Spatial lagged models (SAR) include a parameter of interest on the right hand side of the regression equation, calculated in some studies as the weighted average of alcohol outlets [16,57] or socio-demographics in neighbouring regions, to estimate the incidence crime. Whereas, the spatial error models (SEM) restrict autocorrelation to the error term assuming missing variable bias, effecting the covariance structure of the random disturbance term (e.g., in [57] the spatial error is a random effect) [101,102]. Frequently, spatial models use a contiguity spatial weights matrix to represents the dependency between values or errors at each location and adjacent locations among analysis units, though distance weighted matrices also exist. CAR models, in contrast to SAR and SEM models, assume the state of a particular area is influenced by its neighbours and not neighbours of neighbours (Markov property) applying a symmetric weights matrix.

Pursuant to the spatial models, two cross-section studies explored geographically weighted regression (GWR) [36] or Bayesian spatially varying coefficient process (SVCP) models [35] to estimate how alcohol outlet density influences violent crime across local regional areas. In contrast to the spatial regression models above, spatially varying coefficient models do not assume the relationship between alcohol access and crime is constant across space and instead estimate coefficients for regions across the study area (e.g., census tracts or block groups in these cases). The GWR method fits an linear regression model for each location in the dataset using data collected from a specified radius around the point/region, weighted in varying degrees of importance using a kernel function, such that data further away from the units is less influential that data close by [103]. The “optimal” radius is calculated using cross-validation. In the Bayesian spatial varying coefficient model, random effects (intercept and effect parameters) are defined in the prior and borrow strength from local data exhibiting spatial autocorrelation (defined using contiguity matrix or distance weighted function). The spatially varying coefficient process then uses a prior joint specification of the coefficients that models the spatial correlation of the coefficients as a continuous process (i.e., multivariate conditional autoregressive model) [104], and parameter inferences are possible by sampling the posterior distributions using MCMC sampling.

Panel studies used a variety of fixed and random effects modelling, fit with maximum likelihood (n = 7) or Bayesian (n = 2) estimation, to model how alcohol price [25,58], excise tax [105], and outlets densities [31,57,85,106,107] influenced the incidence of crime over space and time. To ensure cross-sectional and temporal differences did not bias results, researchers applied either space-time fixed [47,105,106] or random [25,57,58,85] effects for units and time periods. In the fixed effects models, researchers considered the lagged effects of alcohol access or socio-demographics over time [105,106] or space [47] on the incidence of crime, but did not explore space-time interaction.

In a more complex panel model, Poisson Bayesian space-time misalignment analysis was conducted to estimate how alcohol outlet density in focal region and neighbouring zip codes (lag) effected the count of assault injuries over 14 years [31]. The Bayesian spatial misalignment model addressed how the geographic delineation of zip codes varied over the study period. The authors specified a CAR random effect for each year’s spatial adjacencies to control the influence on autocorrelation between units. A random county and country level effect were also used to control for the nested structure of the zip codes, and year specific intercepts where implemented to assess statewide changes in assault risk not explained by the neighborhood demographics, alcohol outlet densities, overall hospitalization rates, population density, retail clutter, presence of highways, and ZIP code instability (misalignment) covariates. Successive models were run to explore additional lags and bar interactions effects on crime.

In the less common intervention panel studies (n = 4), applying mixed modelling techniques, researchers contended with space-time effects and monitored if a significant change in the incidence of crime occurred after a change in alcohol outlet closing times [46], allowance of Sunday alcohol sales from packaged retail stores [86], alcohol tax increase [23], or decrease in alcohol outlets [30]. In two cases, fixed effects were used to model the influence space and time units on crime [23,46] though both studies explored temporally lagged influences on crime including: alcohol consumption per capita [23] and change in municipal dry laws [46]. Finally, dummy variables were used to signify if a significant shift in the rate of crime occurred after an alcohol tax change [23] or restricted alcohol outlet closing times [46].

The random effects panel studies (n = 2), applying Bayesian estimation, modelled the change in quarterly count of alcohol-related crashes post lifting alcohol sales ban across 33 counties [86] and the change in probability of assaultive violence after and alcohol licenses surrenders across 480 census tracts [30]. The probability of a crash was estimated using previous quarterly state-rate of crashes, the yearly change in crash rate, socio-demographic controls and a random intercept indicating the change in Sunday sales of alcohol (zero before, mean sales after sales ban). Weakly informative priors were specified for each parameter in the model leaving the posterior inferences largely influenced by the dataset. A CAR model was used to monitor changes in assaultive violence after outlet licence surrender assuming a Poisson distribution for crime data. Alcohol exposure was measured as a dichotomous indicator of census tracts surrendering alcohol licenses, the percent of surrender, alcohol outlet density, and a dual change point interaction term specifying the year and tract. Control covariates included yearly: race, young male population, poverty, and damage per square mile, and a spatial error. The spatial error model accounted for residual similarities across neighbourhoods specifying the prior mean of the error in the focal tract should be equal to the average error in the adjacent census tracts (gamma hyperprior distribution having mean 1 and variance 10 used). All other covariates priors were specified as having a normal distribution centered at 0 with precision 0.00001 (i.e., non-informative). Marginal posterior distributions for all parameters were obtained via Markov chain Monte Carlo (MCMC) sampling.

#### 3.4.4 Regression Trees, Cluster Detection, and Mapping

Pursuant to traditional effects modelling, Multiple Additive Regression Tree method (n = 1) was applied to account for the effects of a percent change in alcohol-license outlets on violent crime rates after multiple alcohol-license surrenders in Los Angles California [108]. Regression trees are a computationally intensive, non-parametric method, of recursively splitting data based on thresholds of the singular variables to maximize the homogeneity within the resulting response groups (e.g., crime rates), using (in some cases) the analysis of variance [109]. The resulting tree shows a hierarchy of selected explanatory variables, and interactions among, though no formal coefficient estimation or significance testing are available [110]. To avoid over fitting and provide a more rigorous evaluation of explanatory variables influence on the model fit, bagging and boosting regression trees ensemble methods were developed. These methods use multiple trees, derived from sub-samples or residual data to predict the response (crime) to stabilize model results [111]. Yu et al., [108] study included a continuous measure of on and off license alcohol outlets densities per square mile from 1990–1999, the proportion of licences surrender after civil unrest, and accounted for spatial structure using a CAR term to explain the variance in violent rates across census tract units of Los Angeles California.

Beyond estimation approaches, alcohol and crime studies have emphasized mapping and graphing as valuable techniques for identifying spatio-temporal patterns between crime and alcohol consumption for policing (n = 5). Space-time mapping was conducted to understand how liquor violations, assaults, batteries, vandalism, and noise complaints emerged through time and space in proximity to the university bar district of Madison Wisconsin [112]. Graphing identified the temporal distribution and proportion of assault per alcohol establishment license type in the Newcastle & Wollongong Australia [113]. Spatial cluster methods illuminated where alcohol outlet densities and crime rates frequencies significantly diverted from an expected random pattern [114]and cellular automata models were used as the first prospective (forecast) analysis to assess how relative risk ratios of crime (crime as a proportion of alcohol density) were expected to disperse with changes in population at risk across a detailed (50m resolution) downtown Vancouver British Columbia study [48].

Specifically, the spatial cluster method identified agglomerations of alcohol outlet densities using an empirical Bayesian rate standardizing scores per roadway mile, and then applied a Moran’s I local analysis to identified block groups (lag 1 contiguity) where the rate of alcohol outlets exceeded the mean overall rates, and significantly departed from what would be expected under a random assignment of alcohol outlets across the study region [115]. Alcohol outlet agglomerations were compared to regional violence counts using a foci cluster test specified as the sum of the differences between observed and expected assault counts at each location weighted by the exposure to alcohol outlet agglomeration. In this sense, the statistic explained a distance decay effect identifying the spatial extent at which the observed number of assaults exceed the expected [114].

Cellular automata methods, similar to agent based modelling, forecasted crime dispersion based on spatial distribution of alcohol outlet seats using a 50m grid across the Vancouver area. Each grid cell was specified with a number of finite states of possible violent crime risk, and a contiguity neighbourhood around each cell was defined. The initial state of each cell was trained by observed alcohol outlet seats and violent crime risk. A new state for each cell was created according to a fixed rule (blocks with high relative risk were specified to increase violent crime frequency) conditional on the current state and of the cells in the adjacent neighbourhood. The simulation was run 2300 times and in each case high risk violent crime blocks multiplied when liquor licenses clustered, creating the first prospective analysis of alcohol exposure and criminal behaviour.

## Discussion

### 4.1 Methods

A large variety of modelling and exploratory techniques were applied to study the effects of alcohol exposure on criminal behaviour (Table 4). Datasets varied in exposure indices and spatial and temporal detail from large state/city/district overviews of crime rate changes after alcohol policy changes [18,23,65,70] to detailed block level analysis of alcohol outlet density and crime clusters [48,114] with each providing unique information for alcohol policy planning. Policy makers are interested in how exposure to alcohol affects overall population rates of crime, while also wanting to address neighbour needs for policing around troublesome alcohol establishments, local zoning policy, or approval of new alcohol establishments [2]. Therefore, estimation and prediction techniques were mindfully selected to provide guidelines for alcohol-crime prevention. We address the strengths and weaknesses of common quantitative approaches, and data collection methods to guide future alcohol-crime research.

**Table 4:** Applied quantitative methods.

Method	Type	Application	Suitable Dataset Structures	Considerations	Applied
ARIMA	Model	Forecasting model used to predict crime-trends (rates or counts) through time. Most often used to understand if the rate or count of crime changed after an alcohol policy intervention.	Times-series	Times-series must be stationary, which can remove information about the temporal patterns of criminal behaviour.	[28,59–62]
GLM	Model	Regression model used to understand how alcohol consumption or alcohol exposure in an area influences crime (rates, counts, odds) across space and or time using fixed effects. Policy interventions were monitored using a dichotomous intervention variable.	Times-series Cross-section Panel	Model residuals must be independent between analysis units (time and or space).	[6,8,10,13,18,20,27,29,44,45,49–56,64–82]
Hierarchical, non-linear models and extensions (GLMM)	Model	Extended regression models used to estimate crime (rates, counts, odds) across space and time as a function of alcohol consumption, access, or other explanatory variables. Effects were random, or mixed, and sometimes hierarchical in structure. Temporally or spatially lagged variables were explored. SAR, CAR, SEM extensions provided useful techniques for modelling spatial autocorrelation across small contiguous unit studies (e.g., census, postal, neighbourhood, block). Policy interventions were monitored using a dichotomous intervention variable.	Times-series, Cross-section Panel	Model residuals must be independent between analysis units (time and or space).	[7,11,16,17,21,23,25,30–34,46,47,57,58,83–100,105–107]
GWR and Bayesian SVCP	Model	Regression models used to specify regional coefficients to address spatial heterogeneity (data relationships that vary across space). Bayesian SVCP method offered a robust statistical estimation, over GWR.	Cross-section Panel(Data must be spatially aggregated to points, grid, or contiguous polygons)	GWR is vulnerable to multiple significance testing. Estimated coefficients should not exhibit positive spatial autocorrelation.	[35,36]
Regression Tree	Non-parametric model	Non-parametric recursive partitioning method used for modelling crime rates or counts as a function of multiple explanatory variables including categorical variables, spatial lagged variables, or CAR terms to model spatial or temporal trends. Policy interventions monitored using dichotomous intervention variable.	Time-series Cross-section Panel	No formal coefficient estimation or significance testing available.	[108]
Cluster Detection (e.g., Local Moran’s I)	Statistical test	Statistical test used to identify (map) areas of high crime or alcohol exposure concentrations.	Cross-section (Data must be aggregated to contiguous spatial units)	User defined spatial weights matrices can influence cluster results. Irregular spatial units can also bias results.	[114]
Cellular automata	Systems model	Discrete model used to predict future crime dispersion based on changes in alcohol exposure using a set of user defined “rules”. The model began with a grid, a fixed state for each cell, and a rule for transformation of the “state” over time.	Cross-section Panel (Data must be superimposed onto a grid)	No formal statistical estimation. System rules (algorithms) are user defined.	[48]
Mapping and graphing	Visual and quantitative method	Used to access if the distribution of crime coincides with alcohol exposure over space and time.	Cross-section Panel	No formal statistical estimation. Limited ability to access multiple effects on the distribution of crime.	[112,113,144]

ARIMA modelling was used for 33% of the intervention time-series studies, with the most recent published in 2012 [28]. While it is possible to use ARIMA models for change point analysis, some limitations exist; namely, the removal of temporal trends and seasonal oscillations in crime during the differencing technique to ensure stationarity among the crime series [63]. By removing information about the timing of crime one is limiting information for crime prevention strategies and information for local alcohol policy/zoning. Further, the structure of the ARIMA model challenges researchers ability to contend with missing data or explore stochastic exploratory effects on the alcohol-crime relationship [116]. Therefore, it is not surprising that 54% of the intervention time-series studies used regression to quantitatively summarize any changes in the incidence of crime post regional alcohol policy changes.

Regression modelling was the most commonly used method of crime estimation (86%) spanning the widest variety of datasets (cross-section, time-series, panel and intervention) and distributional characteristics (Section 3.4.2 & 3.4.3). GLMs (41% of studies) had the advantage of testing constant and seasonal trends on crime, in addition to alcohol exposure, over methods such as hypothesis testing (e.g., chi-square [117,118]). However, there were drawbacks when considering the statistical assumption of: independence between crime measures, correct specification of link and variance functions, little to no multi-collinearity among the explanatory variables, and independent uncorrelated residuals.

A limitation of the intervention time-series studies applying GLMs, without fixed time effects (n = 5), included the assumption that monthly crime data were independent between time periods and any changes in alcohol policy would have an immediate effect on crime. Ignoring implication of time can cause positive serial autocorrelation in errors, and miss any time-lagged effects of the alcohol exposure on crime [119]. Notably, when serial autocorrelation exists between the temporal units the significance of the intervention variable can be overestimated. A small number of the (n = 5) intervention studies did test for data dependence between months [27,65–68] and one corrected for secular and seasonal effects on alcohol consumption and crime [70], though half of the results may have been vulnerable to untested temporal bias. Intervention time-series analysis may be better addressed by mixed modelling approaches incorporating time lagged explanatory variables or structured time series methods that explicitly address trends and seasonality inherent in crime data.

Spatial autocorrelation was an underlying concern for contiguous (n = 10) multi-regional cross-section studies applying fixed linear regression, most often, to study the regional effects of alcohol density on violent crime. Data dependence (positive correlation) between units can lead to correlated residuals, ultimately reducing the standard error and biasing coefficients [38,102]. More recent publications (published since 2011) tested for residual spatial autocorrelation [10,45,75–77] though half did not address the independence assumption. When positive autocorrelation was found, one study remedied significant spatial autocorrelation by removing spatial units instead of applying a more appropriate spatial lag or error model (See Section 3.4.2).

A further concern of the cross-section studies was the application of GLM technique to nested data (n = 7). Nesting often occurred when researchers were modelling the influence of both individuals’ consumption habitats and regional alcohol exposure on the individual level incidences of crime. Without specification of the hierarchies (e.g., generalized linear mixed model) it is possible that correlated errors exist among groups (e.g., dependence between responses pulled from the same regional area/level of alcohol access) which can under estimate standard errors, and in some cases, incorrectly specify the magnitude of the explanatory effects (see [120]).

Panel studies applying fixed effects on the unit and time covariates (n = 4) sacrificed statistical power to avoid omitted variable bias as the degrees of freedom diminish for every space and time unit. The models also become vulnerable to over fitting as the space and time effects are not generalizable to other regions and time periods. Understanding intra space-time patterns is key for alcohol policy planning in an effect to address effects on study applied a jackknifing approach to monitor the impact on the estimated coefficient when one space-time period is left out of the analysis [8]. Researchers would further benefit from the specification of random space time effects, especially with shorter time-periods and a greater amount of spatial units, in order to conserve statistical power [121]. Panel models should also consider the implications of alcohol access in previous units and regions as well as within unit variance.

Hierarchical models (e.g., GLMM, GAM, and Bayesian SVCP) were better suited for estimation when addressing the complex framework of alcohol-crime studies incorporating data from multiple regions and time-periods. Specifically random effects modelling permitted the influence of explanatory variables to fluctuate over space and time (random slope or intercept model) and can be keenly useful when addressing spatial variation in the expected outcome [121]. For example, you can condition the estimated value of crime toward the mean for regions with fewer persons instead of predicting extremely low numbers [121,122], particularly useful for small scale regional modelling (e.g., census, neighbourhood, blocks). Further, hierarchical models can estimate the effects of explanatory variables on crime measured at multiple scales allowing researchers to consider direct factors (e.g., alcohol blood level) and environmental effects (e.g., regional alcohol outlet density, or demographics) on the incidence of crime. Mixed modelling also offer approaches for modelling autoregressive processes (lagged or spatial error models) when the space and time detail in data increase such that researchers have to consider the effects of alcohol access in previous time periods or neighbouring units on the incidence of crime. Alcohol consumption in one neighbourhood can lead to crime in an adjacent or further region and changes in alcohol policy may have a delayed influence on crime incidence as the population recognizes modifications, increasing the importance of considering lagged variables.

Bayesian estimation also provided flexible inference methods for modelling hierarchies [32,84] space [31,35], and space-time [30,85,86] dynamics. Improving upon frequentist techniques, Bayesian Spatially Varying Coefficient Process offer inference possibilities for modelling non-stationary datasets (controlling for correlation among regionally estimated regression coefficients), in contrast to GWR models which use an iterative algorithm lacking formal statistical properties of inference [104]. Because of these advantageous we are seeing a recent trend in the publication of Bayesian inference across the alcohol-crime literature (seven published since 2007) most likely influenced by the increasing hierarchical and space-time detail of data and free software (e.g., WinBugs) for model fitting and computationally intensive sampling of the posterior distributions for estimation.

In addition to mixed modelling techniques, we also see utility in the less common applied exploratory methods, specifically cluster detection and density mapping, which can illuminate specific risk locations of alcohol-related crime [123]. Cellular automata also poses an alternative prospective modelling approach where known information about alcohol exposure and crime can train a computation model to predict where crime will lead in future scenarios of exposure [48]. However, these methods lack tradition coefficient estimation, statistical significance testing, and limit the ability to study simultaneous effects on crime. Parameters are often specified by the user (e.g., cellular automata “rules” and weight matrices in cluster detection) introducing user bias. What they do provide is local specification of high risk areas for policing and regional planning, and unique methods for predictive simulations when alcohol exposure increases (e.g., additional retail stores, on-premises drinking establishments, or extended hours of sales).

### 4.2 Future research

Pursuant to modelling considerations we found the practice of standardizing crime counts by residential population data predominant across rate calculations. It is likely when using smaller geographic units (blocks or neighbourhood) for analysis the residential population is unrepresentative of population at-risk [43] displacing the true spatial pattern of crime [124] thereby altering model results for small areas studies. For example, people living in an area are not necessarily the population consuming alcohol and committing crime. Often establishments that sell alcohol draw people from neighbouring regions to their premises altering the population at risk in time and space [125]. Depending on the study, using residential population counts can skew the relative risk scenario of crime and alter relationships estimated in models applying residential population rates, especially across smaller areas such as blocks, census tract, or neighbourhoods where persons could readily venture between. Opportunities exist to use ambient population data (data representing the spatial and temporal fluctuations of populations). Products such as Landscan Data (1km resolution, http://web.ornl.gov/sci/landscan/) or social media crowd sourced data [124] can be used to gain dynamic population estimates for improved rate calculations and provide population information for retrospective and prospective modelling. Landscan data redistributes residential population counts, using complex land use models, to identify where persons are mostly likely to spend their time in a 24hr period. Whereas, social media demographic estimates pin media users to the geographic location in time showing demographic variances across space and time, and are likely to represent the younger drinking population vulnerable to nuisance and assault crimes [2,126,127].

Emphasis on the accurate measurement of alcohol exposure is also prudent with the majority of analysis (89%) inferring causation using exposure measures (alcohol outlet density, hours of sales, alcohol sales volumes, and trading hours) instead of direct consumption information (blood alcohol level, drinking frequency). Generally, we found studies exploring direct alcohol consumption indicators (blood alcohol level and consumption patterns) identified positive significant results between alcohol consumption and crime [6,13,23,29,44,54,55,78,79]. Whereas studies using alcohol exposure measures such as alcohol sales lock-outs [18,65], change in the hours of sales [62,66–69], change in establishment hours [118], modification of alcohol tax [60,61,82,105] or alcohol outlets (measured at the municipal level) [89,91] found no significant relationships. Insignificant findings, were exclusive to time-series [60,61,65,67–69,118], cross-sectional [89,91], and panel [82,105] assessments aggregating data to large spatial units such states (n = 1), countries (n = 5), cities (n = 3), or municipalities (n = 2), which may indicate that both the type of index and level of spatial aggregation can mask effects. Overall the choice of scale is limited to available data and we can not make conclusions across scales, though improvements can be made to alcohol exposure measures. Simply the difference in measurement of alcohol outlet density per region is one example. Regions of equal outlets and populations can have vastly different access if spread across difference sized areas. Similarly standardizing by area does not represent the paths people readily use for travel. While most studies standardize outlet density by population per region [34] or per area [128], roadway standardization is regarded as a preferred method of representing “access” [88].

Further, many studies combine alcohol establishment types to model the association between crime and indicators of consumption [6,8,29,35,90–92,95,129]. However, it has been established that specific establishments types contribute disproportionately to increasing the rates of crime [113,130]. To illuminate these connections researchers need disaggregate alcohol establishments, especially across small unit studies were correlation among densities is less likely. Additionally, researchers could explore attributing density measures with seating capacities as not to treat each establishment as having an equal weight on consumption patterns [48]. Only one study represented on-premises alcohol outlet density using seats [48].

We recognize the limitations surrounding the level of spatial and temporal detail available for crime models using traditional data sources, such as aggregated police data or government records. We see utility in assessing if social media can be used to track alcohol consumption and crime patterns in space and time by searching user’s messages on twitter feeds. The information content provided by social media is being utilized in health research [131], and could prove resources for crime and alcohol studies. Participatory mapping, where respondents connect responses in space and time on a geographic interface, could also become a more common application across the consumption and crime surveys to source information about the probable locations of alcohol consumption and witnessed alcohol-attributable crime. The advantages of participatory data collection for health research are well known [132,133], but have not extended to crime-alcohol modelling.

Regarding dataset structures, the robustness of cross-sectional studies (n = 43) could be improved by increasingly collecting data that link intoxication level and the place of last consumption, to the location of the crime. For example, in studies, such as Chikritzhs & Stockwell [44] and Macdonald et al. [78], researchers analyzed crime records in conjunction with blood alcohol levels and place of last consumption. These studies are well poised to draw hierarchical connections between individuals and environmental influences on drinking patterns and subsequent criminal behaviour. Other researchers applied a local-level analysis to gain insights on the frequency of crime in and around alcohol establishment’s linking offences with the types of alcohol outlets [18,26,72,113]. Studies connecting consumption to specific locations are invaluable crime prevention, but scarce across the literature.

When smaller regions are studied, heath researchers have noted the distance effects of alcohol availability within regions on neighbouring regions’ crime rates, and these influence have been observed by fitting models with spatially lagged variables where within each spatial unit crime rates are predicted by establishments within neighbouring areas [16,57]. Rarely in these studies are the effects explored outside of the adjacent areas (e.g.,[31,96]); however, as the spatial units become smaller (i.e., blocks) the movement of intoxicated individuals across more than one spatial unit is likely and researchers should address the distance at which the effect is negligible by using multiple spatial lags within small area studies.

To date, explicitly addressing the concept of proximity between alcohol establishments and crime is limited [20,114]. With advances in technology for mapping alcohol establishment and crimes, it is possible to address the diffusion of crime around each establishment in space and time. Using distance decay functions [134–136], parameters can be quantified to explain the expected frequency of crime as a function of distance by treating alcohol establishment locations (or clusters) as the origins of crime. Space-time bivariate point pattern analysis [136] can also statistically assess the spatial extent (i.e., radius) crimes cluster around outlets. These results would provide evidence based information for setting restrictions on the proximity of alcohol establishments in an area. Only one known study has explored bi-variate Ripley’s k-function to determine the distance at which point level crime data clusters around alcohol establishments [137] and additional analysis is need to understand if these distance thresholds are consistent across study areas for implementing policy.

Mapping has been largely overlooked analysis strategy, likely because of the privacy concerns associated with crime data. Out of the 90 studies summarized, 18 mapped the distributions of alcohol access and/or crime, and to a lesser extent fitted model values or errors [30,108,138]. However, maps can illuminate data outliers and applicable spatial scales for model assessment. Criminologists to date have had a vested interest in understanding the frequency of crime through space and time and studies have been conducted to address the stability of crime hot-spots [139,140]. In the cases where alcohol establishment densities remain static, it is still useful to study how crime hot spots emerge through hours of the day around these establishments. To observe how clusters of crime form over time three dimensional kernel density mapping [141], or scan statistics [142] are possible approaches, providing a novel and interesting perspective for alcohol policy literature.

Identifying thresholds at which alcohol access substantively increases crime rates is also an interesting avenue of future studies. Both Livingston [11] and McKinney et al. [51] observed that violent crimes exponentially increased when the count of alcohol establishments met or exceeded 25 units per postal or zip code. These findings signify a change in the environment, merging from community areas to entertainment districts. To understand if these thresholds are cross-regionally or cross-culturally stable, it is of interest for criminologist and health researchers to employ modelling techniques that can accommodate non-linear response relationships, either in the form of transformed specification before modelling (i.e., GLM, GLMM), or non-parametric methods such as regression trees [109].

## Conclusion

Study designs and statistical approaches characterizing the relationship between crime and alcohol are best chosen based on the research question and nature of data. Researchers studying the influence of alcohol exposures on the rate or count of crime over large areas using multiple spatial units (census tracts, neighbourhoods, blocks) will likely turn to spatial regression, hierarchical models, and spatial varying coefficient models to capture spatial effects. While, crime data collected over areas considered to be demographically homogenous will mostly likely apply time-series analysis to understand how alcohol policy affects crime over larger population groups. Novel sources of spatial data are going to create further opportunity to utilize non-traditional methods to study how the size and capacity of drinking establishments impacts consumption and ultimately crime, across space and through time. There are new techniques available for rate calculations across small analysis units, and we anticipate a surge in the spatio-temporal analysis of the alcohol consumption and crime connection. There is a need to inform policing and alcohol policy by identifying how consumption in specific locations influences regional crime. With advances in spatial-temporal data collection we expect a continued uptake of flexible Bayesian inference, greater inclusion of spatio-temporal point pattern analysis, and prospective modelling over small areas.
